# Childhood Neurogenic Stuttering Due to Bilateral Congenital Abnormality in Globus Pallidus: A Case Report and Review of the Literature

**Published:** 2016

**Authors:** Mohammad Javad SAEEDI, Ebrahim ESFANDIARY, Mostafa ALMASI DOOGHAEE

**Affiliations:** 1 Department of Anatomical Sciences and Molecular Biology, Isfahan University of Medical Sciences, Isfahan, Iran; 2 Isfahan Neurosciences Research Center, Alzahra Hospital, Isfahan University of Medical Sciences, Isfahan, Iran; 3 Department of Neurology, Iran University of Medical Sciences, Tehran, Iran

**Keywords:** Neurogenic stuttering, Basal ganglia, Globus pallidus

## Abstract

**Objective**

The basal ganglia are a group of structures that act as a cohesive functional unit. They are situated at the base of the forebrain and are strongly connected with the cerebral cortex and thalamus. Some speech disorders such as stuttering can resulted from disturbances in the circuits between the basal ganglia and the language motor area of the cerebral cortex. Stuttering consists of blocks, repetitive, prolongation or cessation of speech. We present a 7.5 -year-old male child with bilateral basal ganglia lesion in globus pallidus with unclear reason.

The most obvious speech disorders in patient was stuttering, but also problems in swallowing, monotone voice, vocal tremor, hypersensitivity of gag reflex and laryngeal dystonia were seen. He has failed to respond to drug treatment, so he went on rehabilitation therapy when his problem progressed. In this survey, we investigate the possible causes of this type of childhood neurogenic stuttering.

## Introduction

Stuttering includes of disorders in rhythm of speech, tension in laryngeal and pharyngeal muscles and uncontrolled, repetitive, prolongation or pause of a voice (1). Stuttering is a disorder of communication and is associated with increased incidence of mental health problems and decreased quality of life (2). Researches about the nature of stuttering have produced an extensive amount of data during the past years, but the mechanisms behind the speech disturbances and the speech initiation problems are still not clear (3).

The basal ganglia are the largest subcortical structures in the human forebrain, placed in a fundamental position to influence motor behavior, emotions, and cognition (4, 5). The main components of the basal ganglia are the caudate nucleus, lentiform nucleus, substantia nigra, the subthalamic nucleus and claustrum. The lentiform nucleus lies lateral to the internal capsule and divided into a lateral part (the putamen) and medial part (the globus pallidus) (GP). 

The GP is further subdivided into internal and external segments (6). GP is one of the main components of the motor circuit of basal ganglia and affect the cerebral cortex output via connections with putamen, subthalamic nucleus and thalamus (direct and indirect motor circuit).

The most common cause of neurogenic stuttering (NS) is brain lesion (7). General features of NS are: A) Principal feature is repetition of syllable and sound blocks are less frequent; B) Dysfluencies occur on structural words closely as frequently as on substantive words; C) The stutter might be bothered, but does not appear anxiety; D) Stuttering features (e.g., Repetitions, prolongations, and blocks) do not occur only on initial syllables of sentence; E). Secondary behaviors (e.g., eye blinking, head nodding, etc.) are not related with moments of disfluency; F) No adaptation effect exists; G) Stuttering happens consistently across speech tasks of various forms; H) Generally, patients have additional signs of aphasia or dysarthria (8).

This paper deals first with the report of NS with bilateral congenital anomaly in GP, followed by description and investigation of possible causes of this type of stuttering.

## Case Report

**Background and preliminary physical examination:**

A 7.5–yr-old ambidextrous male child was referred by multiple problems such as balance and gait disturbances, incoordination of extra ocular muscles when walking, disorders of gag reflex and swallowing, vocal tremor, monotone speech and weakness of mastication muscles. According to his mother, walking of the patient was delayed.

Level of plasma copper was in normal range (90 Mic g /dl) and there was no noticeable increase or decrease in plasma lactate (9.6 mg / dl). His cranial nerve examination revealed that response of pupil to light and accommodation was normal. Fields of vision were full to confrontation testing. There were no abnormalities on funduscopic test. The Kayser–Fleischer ring not appeared around the corneo-scleral junction. Deep tendon reflexes were exaggerated with pattern of upper motor neuron disease. Bilateral Babinski’s reflex were abnormal (Babinski+). Among neonatal reflexes, biting reflex was remained. According to lack of The Kayser– Fleischer ring and normal range of cooper plasma level, Wilson’s disease in patient was rejected. 

Among all signs of speech disorders, stuttering was obvious. The traces of witnessed stuttering have been discerned since the patient has been 4 yr old. 

**Fig 1:**
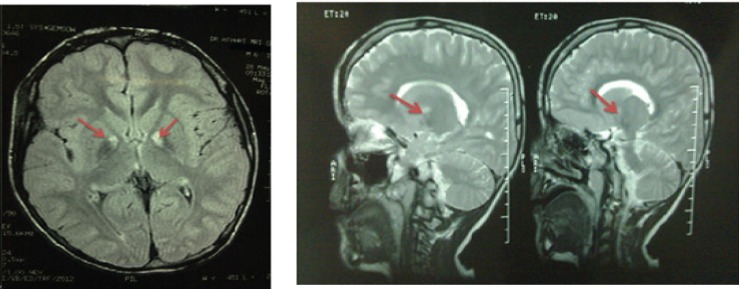
Magnetic resonance imaging (MRI) scans of patient showing bilateral Globus pallidus (GP) abnormality (red arrow). Left: Axial FLAIR brain MRI, shows hyposignality in bilateral GP with a hypersignality in its medial part; Right: Sagittal T2-weighted brain MRI shows hypersignality in medial part of GP

There was no stuttering in the patient family history but Patient’s grandfather suffered from Parkinson’s disease in late period of his age. No point of delay delivery or cyanosis after birth was found. The visiting psychologist did not report any mental disability in child. Para clinical diagnosis such as auditory brain stem response (ABR), computerized tomography (CT) scan and electroencephalogram (EEG) did not show any lesion in patient’s nervous system. Finally, for definitive diagnosis, magnetic resonance imaging (MRI) was ordered for the patient and MRI showed bilateral globus pallidus lesions (Figure 1).

**Neurological examinations associated with the speech**

The general sense of tongue epithelium in territories of trigeminal and glossopharyngeal sensory branches nerves was normal. The tongue sense of taste in territories of facial and glossopharyngeal nerves was also normal. Oral tactile agnosia was not shown in patient. The movements of tongue were intact, but the rate of consecutive movement was slow (hypoglossal nerve test). Patients’ hearing was normal and the result of ABR test confirmed our assessment (test of cochlear part of vestibulocochlear nerve). The ability to cough in the child was normal (vagus nerve test). The hypersensitivity of gag reflex was shown. Mastication muscles were weak (test of motor division of trigeminal nerve). The movements of facial muscles were normal (test of motor division of facial nerve).

**Neurolinguistics and Stuttering Examinations**

The patient was aware and exhibited normal behaviors. Upon language skills assessment, he had normal understanding, naming and repetition. The scarcity of language disorders has been discerned by unofficial tests and counsels of speech and language pathologists. 

For definitive diagnosis of stuttering, we examined the patient speech with neurolinguistics tests. 

Neurolinguistics and stuttering tests are as follows: A) Counting 1-10: shown the loss of automatic series, many hesitations, pauses and slow speech; B) Reciting the days of the week: hesitations and abnormal pauses were present; C) Speech and non-speech Consecutive movements: repetition of /a/ and Opening and closing the mouth, the rate of movement was very slow; D) The presence or absence of physical tensions or secondary behaviors (e.g., eye blinking, head nodding, etc.) associated with dysfluency: these behaviors were seen; E) Exhibiting negative and aggressive reactions toward his dysfluency: the patient exhibited these reactions (e.g., Refuse to answer); F) Whispering speech: there was no stuttering observed; G) In his spontaneous speech and dialogue, blocks and abnormal pauses were present; H) During Singing, the numbers of dysfluency of speech were decreased; I) After several time of repetition of one sentence, the fluency of speech was increased; J) Upon language assessment, the patient had normal understanding, naming and repetition; K) Language formulation abilities were grammatical and correct, with no evidence of word finding difficulty or childhood aphasia. 

For investigate the severity of stuttering, we used stuttering severity instrument-3 (SSI-3) score. The stuttering severity of patient was moderate (SSI-3 score: 23). In sum, when compared to the criteria usually accepted to describe stuttering, the patient’s speech profile did conform with many those characteristics. 

## Discussion

NS has been reported following lesions in almost all parts of the human brain, except the occipital lobe (9). 

Published medical reports describe that the frontal lobe, the thalamus, and the basal ganglia are commonly involved in NS (3). Lesions of primary language cortex would not produce stuttering (7). The basal ganglia are involved in several facets of psychomotor behavior. Anatomically, this group of nuclei is mainly involved in a closed cortical- basal ganglia-thalamuscortical loop (10). The newly literature provide valuable understanding on how the direct and indirect pathway of motor loop of basal ganglia encodes action sequences (11). The speech and skeletal motor systems have common neural control mode despite major biomechanical differences (12).

Regarding, the basal ganglia, perhaps the strongest proof for involvement of basal ganglia in speech dysfluencies is associated with stuttering comes from pharmacological studies (13). In the human brain, the striatum receives the rich dopamine innervation, and drugs such as haloperidol that block type D2 dopamine receptors have been effective in transient treatment of stuttering (3). The main role of basal ganglia in NS is also supported by neuroimaging surveys. Involvement of many parts of basal ganglia in stuttering has been reported (7).

In non-stuttering individuals, there are positive feedbacks between Broca’s area in the left inferior frontal gyrus and speech motor regions (14). In stuttering conditions, the striatum part of basal ganglia would then receive unsuitable input from the motor cortex, imprecise with respect to both timing and phonological aspects of speech. This inappropriate input could result in a diffuse activation of the striatum possibly associated with beginning of stuttering (14, 15).

Most of the striking area of over activity in patients who stutter is parts of basal ganglia with reciprocal connections with internal GP. Bedsides, stuttering is a disorders associated with disruption in the cortical and subcortical motor circuit (16). Niels et al. also presented two cases of acquired stuttering after deep brain stimulation of the internal GP for treatment of dystonia (17). Dietz et al. investigate the effects of deep brain stimulation of GP on verbal fluency performance and found that stimulation region within the GP did not affect verbal performance in patients who received GP deep brain stimulation (18). In our patient, based on MRI report, there is hypersignality in medial part of GP.

Involvement of the basal ganglia in stutter is much more in the shape of physiologic complications than to be anatomic, for example increase or decrease in the level of the activity of ganglia, and the effect of this topic on the cortex output, has the parallel concept. In case of mentioned disease, there was a significant damage perceived in the MRI, which was more probable to be a neurogenic stuttering, not a developmental one. 

This patient referred to rehabilitation part of hospital by multiple problems accompanied by speech dysfluency associated with stuttering. According to literature, definitive diagnosis of NS is difficult. There were some limitations to this case study. One feature of NS is lack of anxiety, but in our patient aggressive reaction and anxiety following blocks in speech were seen. In NS, secondary behaviors were not associated with moment of stuttering, but in this case, with beginning of dysfluency these behaviors were also seen. NS is usually accompanied by aphasia or dysarthria, but in our patient, there were not language disorders and palsies.

As it has been deliberated earlier; the discussed patient does not possess of all manifestations of a neurogenic stuttering; i.e., some peculiarities of a developmental stuttering have been eloquently handy to descry. In contrary, the prodigious erosion of the basal ganglia and in the other hand pediatric neurologist verdicts bespeaks a neurogenic stuttering. Anyhow, the discrimination of a neurogenic stuttering is solely toilsome. 

**In conclusion, **this patient has a congenital NS but not all characteristics of NS exist in presented case. Because of their association with cerebral cortex, especially with Broca’s area in the left inferior frontal gyrus, the basal ganglia can influence the motor features of speech. Probably in the present patient, disturbances in the circuits between the basal ganglia and the language motor area of the cerebral cortex resulted in motor speech disorders (7, 19).
